# Barriers to adherence to cervical cancer screening care in Northern Tanzania

**DOI:** 10.1093/oncolo/oyaf111

**Published:** 2025-05-16

**Authors:** Tecla Lyamuya, Bariki Mchome, Clara Yolanda Stroetmann, Rogathe Machange, Muluken Gizaw, Rahel Alemayehu, Adamu Addissie, Pendo Mlay, Alex Mremi, Eva Johanna Kantelhardt, Blandina T Mmbaga

**Affiliations:** Department of Obstetrics and Gyneacology, Kilimanjaro Christian Medical Centre, Moshi, Tanzania; Department of Obstetrics and Gynaecology, Kilimanjaro Christian Medical University College, Moshi, Tanzania; Global Health Working Group, Institute for Medical Epidemiology, Biometrics and Informatics, Martin-Luther-University, Halle-Wittenberg, Germany; Department of Obstetrics and Gyneacology, Kilimanjaro Christian Medical Centre, Moshi, Tanzania; Department of Obstetrics and Gynaecology, Kilimanjaro Christian Medical University College, Moshi, Tanzania; Global Health Working Group, Institute for Medical Epidemiology, Biometrics and Informatics, Martin-Luther-University, Halle-Wittenberg, Germany; Department of Obstetrics and Gyneacology, Kilimanjaro Christian Medical Centre, Moshi, Tanzania; Department of Obstetrics and Gynaecology, Kilimanjaro Christian Medical University College, Moshi, Tanzania; Kilimanjaro Clinical Research Institute, Moshi, Tanzania; Global Health Working Group, Institute for Medical Epidemiology, Biometrics and Informatics, Martin-Luther-University, Halle-Wittenberg, Germany; Department of Epidemiology and Biostatistics, School of Public Health, College of Health Sciences, Addis Ababa University, Addis Ababa, Ethiopia; Department of Epidemiology and Biostatistics, School of Public Health, College of Health Sciences, Addis Ababa University, Addis Ababa, Ethiopia; Global Health Working Group, Institute for Medical Epidemiology, Biometrics and Informatics, Martin-Luther-University, Halle-Wittenberg, Germany; Department of Epidemiology and Biostatistics, School of Public Health, College of Health Sciences, Addis Ababa University, Addis Ababa, Ethiopia; Department of Obstetrics and Gyneacology, Kilimanjaro Christian Medical Centre, Moshi, Tanzania; Department of Obstetrics and Gynaecology, Kilimanjaro Christian Medical University College, Moshi, Tanzania; Department of Obstetrics and Gyneacology, Kilimanjaro Christian Medical Centre, Moshi, Tanzania; Department of Obstetrics and Gynaecology, Kilimanjaro Christian Medical University College, Moshi, Tanzania; Global Health Working Group, Institute for Medical Epidemiology, Biometrics and Informatics, Martin-Luther-University, Halle-Wittenberg, Germany; Global Health Working Group, Institute for Medical Epidemiology, Biometrics and Informatics, Martin-Luther-University, Halle-Wittenberg, Germany; Department of Gynaecology, Martin-Luther-University, Halle-Wittenberg, Germany; Department of Obstetrics and Gyneacology, Kilimanjaro Christian Medical Centre, Moshi, Tanzania; Department of Obstetrics and Gynaecology, Kilimanjaro Christian Medical University College, Moshi, Tanzania; Global Health Working Group, Institute for Medical Epidemiology, Biometrics and Informatics, Martin-Luther-University, Halle-Wittenberg, Germany; Kilimanjaro Clinical Research Institute, Moshi, Tanzania

**Keywords:** cervical cancer screening, follow-up, recurrence, suspicious cervical lesion, adherence

## Abstract

**Background:**

Cervical cancer disproportionately affects women in low- and middle-income countries compared to those in high-income countries because of the difference in quality and effectiveness of cervical cancer screening programs. An essential part of effective cervical cancer prevention is the continuum of care for a woman with a suspicious cervical lesion (SCL) consisting of appropriate treatment and, in Tanzania, a follow-up screening one year after treatment. This study aimed at identifying factors associated with non-adherence to the scheduled follow-up after treatment of a SCL. Additionally, the cervical cancer screening results one year after treatment were evaluated.

**Methods:**

A total of 219 clients treated for a SCL between 2017 and 2021 from 8 centres in the Kilimanjaro region were interviewed. Contact and medical information of the clients was obtained at the facilities. Additionally, 11 in-depth interviews with healthcare providers were conducted.

**Results:**

In the quantitative study, 143 (65.3%) clients treated for suspicious cervical lesions adhered to the recommended follow-up appointment. Significant factors associated with poor adherence were individual barriers such as failure to understand why they should return and access barriers to the health facility. The health workers mentioned a lack of awareness and financial challenges regarding transportation.

**Conclusion:**

The complete journey of high-risk women needs attention, otherwise the primary screening will not be effective. Additional efforts are needed to address knowledge gaps and socio-economic problems during the follow-up.

**Operational definitions**

Adherence refers to all women who kept their planned follow-up appointment or came within 30 days of the scheduled appointment.

A suspicious cervical lesion referred to documentation of an aceto-white lesion or an abnormal cytology result using a Pap smear in the registry.

**Implications of practice**

This study shows that a considerable proportion of women fail to return for follow-up after one year. Obstacles include lack of information and lack of understanding on the need for follow-up. This could be tackled by in-depth counseling during the first visit as well as reminders closer to the follow-up appointment. The findings of this study may be used by policymakers and program managers to address obstacles and to develop effective interventions to solve problems related to non-adherence after treatment of a SCL.

## Introduction

Cervical cancer is one of the most frequently occurring cancers in women worldwide.^1^ It is preventable if diagnosed and treated early by adequate cervical cancer screening programs.^2^ In low- and middle-income countries the burden of cervical cancer is larger than in high-income countries^3^ -mainly due to the lack of effective screening programs and low awareness concerning the disease and its prevention measures (Mchome et al, 2020).^4^

In Tanzania, the first screening program was implemented in 2004. The Tanzanian cervical cancer screening guideline advocates for yearly screenings of women living with HIV and once in 3 years for the general population using either VIA or Pap smear. Patients with suspicious cervical lesions (SCL) should receive treatment in the form of cryotherapy, thermocoagulation, or loop electrosurgical excision procedure (LEEP). If possible, treatment should be provided in a single-visit-approach (SVA) meaning right after the initial screening. One year after treatment, all patients are required to return for a follow-up to check for a recurrence or persistence of the lesion.^5^

This study intended to explore the challenges within the continuum of care in cervical cancer prevention by examining the pathways of women with a SCL. It focused on the factors affecting adherence to the recommended one-year follow-up screening after primary treatment for a SCL and the recurrence or persistence of a SCL during the follow-up screen. As the continuum of care is key to a successful screening program, our insights may help enhance the quality of cervical cancer screening programs in Tanzania and other low- and middle-income countries.

## Methods

### Study setting

This study combined qualitative and quantitative methods to examine the continuum of care in cervical cancer screening in 8 health facilities in the Kilimanjaro region. Kilimanjaro is one of Tanzania’s 31 administrative regions, with a population of 1,861,934.^6^ It has a total of 53 health facilities providing cervical cancer screening and treatment of SCL. The participants in this study were recruited from a tertiary hospital, a regional hospital, and 6 health centers.

### Data collection

The data were collected between November 2022 and April 2023. Contact information for all the women with a SCL was taken from the cervical cancer screening logbooks. Women with a SCL were called and asked to participate in a questionnaire-based phone interview. The participants were considered unreachable after more than 3 phone calls were made without success. The questionnaire included sociodemographic characteristics, details of treatment provided for the suspicious cervical lesion, and perceived barriers or enablers to follow-up attendance. This questionnaire had previously been used in a study done in Ethiopia.^7^ For use in Tanzania, we translated it to Kiswahili.

For the qualitative part, we conducted in-depth interviews (IDIs) with 11 nurses who provided cervical cancer screening in the included health facilities. Open-ended questions were used to obtain information on facility-related barriers to follow-up after primary screening and treatment, which were based on a study conducted in Cameroon.^8^ All interviews were conducted in Kiswahili, audio-recorded, transcribed, and then translated into English.

### Data analysis

For data analysis, questions on barriers were grouped into individual barriers and health facility barriers. The questions compiled under individual barriers included: fear of an adverse outcome, fear of the screening procedure, not knowing why they should return for follow-up, forgetting the appointment, not finding it easy to access the health facility, lack of transportation money, lack of time to return for screening, lack of support from their companions, and the preference for other methods of healing such as spiritual or traditional methods. The questions compiled in the health facility-related barriers group included: not receiving counseling, not being counseled on timing, a long wait time, being unhappy with staff behavior, service not being available, and other challenges. Each factor was assigned a positive or negative value depending on whether it was a barrier or enabler. The total barrier score for each participant was calculated. Those scoring equal to or greater than the median were categorized as high and those below the median as low. (Supplementary Table S1).

The quantitative data were analyzed in STATA (Version 15). Logistic regressions were used to determine the association of the study’s variables with the outcomes. For the IDIs, the translated transcripts were imported to NVivo software for coding and generation of themes using thematic content analysis techniques.

### Ethical considerations

Permission was obtained from the Regional Medical Officer of the Kilimanjaro region and thereafter from the College Research Ethical Review Committee. The study participants’ consents were obtained prior to data collection.

## Results

**Figure 1.**
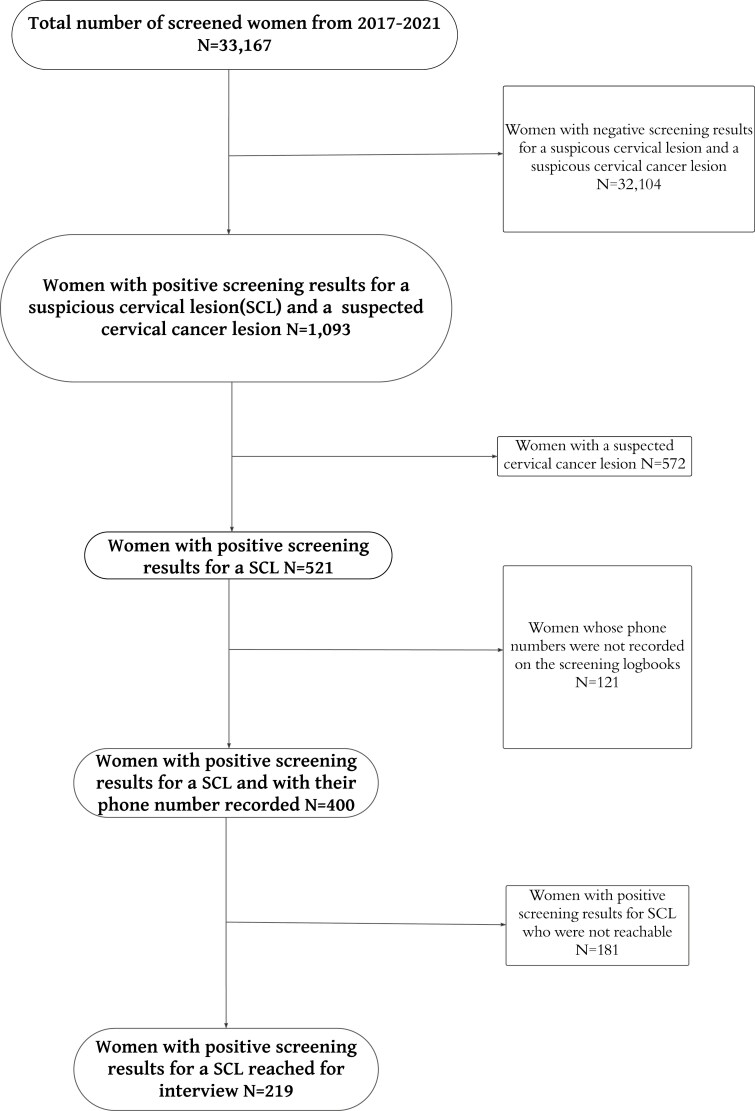
Participants enrollment flowchart.

Between 1 January 2017 and 31 December 2021, 33,197 women were screened in the 8 health facilities. Of the screened women, 521 (1.6%) had a SCL, and 572 (1.7%) were suspected to have cervical cancer. Of those with a SCL, 400 had their phone numbers recorded and 219 were reachable and therefore included in this study (Figure 1). 181women with SCL were not reached and therefore were termed as lost to follow-up.

### Sociodemographic and clinical characteristics of the interviewed patients

The average age of the 219 study participants was 45 years (SD = 11.5); 144 (65.8%) women had primary education; 131 (60.1%) were married or cohabiting; 113 (52.3%) were unemployed, and most (*n* = 90; 56.3%) had an income of less than $61 USD per month. Regarding clinical characteristics, 211 (96.3%) had given birth, with a median of 3 births; 148 women (69.2%) had their first sexual intercourse at 19 years or older, and 135 (61.6%) were HIV-negative. A total of 139 (64.4%) were treated by cryotherapy and 104 (47.5%) of the treated women adhered to the SVA (Table 1).

**Table 1.** Distribution of the participants’ socio-demographic and clinical characteristics.

Variable	Frequency	Percentage
**Age**		
18-29	16	7.3
30-50	142	64.8
>50	61	27.9
**Education level**		
Primary education	144	65.8
Secondary education	46	21
Higher education level	29	13.2
Occupation*		
Un- employed	113	52.3
Business	76	35.2
Employed	27	12.5
Marital status*		
Married/ cohabiting	131	60.1
Single	25	11.5
Divorced	22	10.1
Widow	40	18.4
Monthly income USD*		
No income	16	10
< 61	90	56.3
61-194	33	20.6
>194	21	13.1
**Ever given birth**		
Yes	211	96.3
No	8	3.7
Parity*		
0	8	3.7
1-3	111	51.6
4-5	76	35.4
6 or more	20	9.3
Sexual debut*		
12-18	66	30.8
≥19	148	69.2
**HIV status**		
Positive	76	34.7
Negative	135	61.6
Unknown	8	3.7
Treatment*		
Cryotherapy/thermal ablation	139	64.4
LEEP	77	35.7
**Days elapsed from screening to treat**		
Single visit approach	104	47.5
See and treat	115	52.5

*Frequency does not tally due to missing variables.

Abbreviations: IQR, interquartile range; LEEP, loop electrosurgical procedures; SD, standard deviation; USD, United States Dollar.

### Level of adherence to follow-up and the clinical cervical status one year after treatment

**Figure 2.**
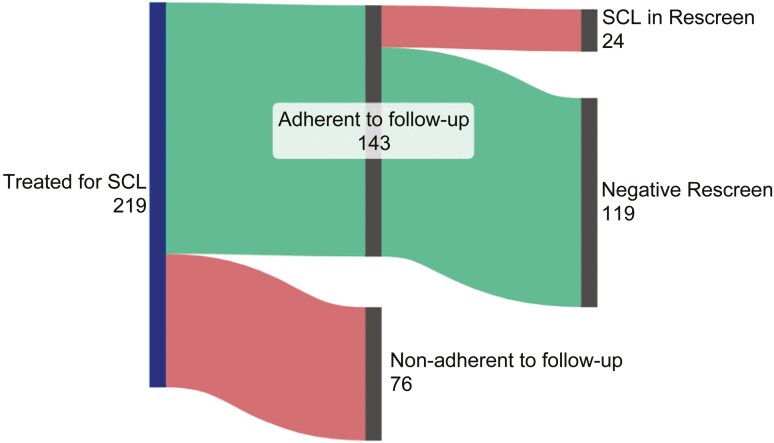
Adherence of study participants to one-year follow-up screening (*N* = 219).

Of the 219 interviewed women treated for SCL, 143 (65.3%) adhered to their recommended follow-up, while 76 (34.7%) did not (Figure 2). Subsequently, among those 143 women, 119 (83%) had negative results, and 24 (17%) had positive re-screening results.

In the IDIs, some of the health care providers perceived the level of adherence to the recommended follow-up to be good: *“A large percentage of them return to the clinic; some forget, and may forget within months, but a large percentage do return.”* Others disagreed: “*To be honest, the follow-up rate is not good. It is quite poor, in my understanding. Since I started working here and with the patients we have assessed and treated, their follow-up is not satisfactory.”*

### Factors associated with adherence to follow-up after primary screening

Adjusted for other variables, individual barriers, occupation, and type of treatment were significantly associated with follow-up adherence. Women who scored high on individual barriers had 0.34 times lower odds of adhering to follow-up as compared to those who scored low (AOR = 0.34; 95% CI [0.15-0.69]). The most common individual barriers limiting adherence among clients were “not knowing why they should return for follow-up” and access barriers to the facility (Supplementary Table S2). Compared to those who were not employed, women who were employed were less likely to adhere to follow-up (AOR = 0.22; 95% CI [0.06-0.73]). Women who were treated by LEEP had lower odds of adherence to follow-up compared to those treated by cryotherapy/thermal ablation (AOR = 0.46; 95% CI [0.21-0.99]) (Table 2).

**Table 2.** Factors associated with adherence to follow-up after one year among women after positive primary screening (*n* = 187).

Variable	Total	Adjusted	
		AOR(95%CI) adherence to follow-up	*P*-value
**Individual related barriers**			
Low individual-score	125	1	
High individual-score	62	0.34 (0.15-0.69)	<.001
**Health facility-related barriers**			
Low health facility-score	122	1	
High health facility-score	65	0.33 (0.56-2.17)	.78
**Age**			
18-29	14	1.14 (0.27-4.86)	.86
30-50	121	1	
>50	52	0.46 (0.20-1.04)	.06
**Education level**			
Higher education level	27	1	
Primary education	120	1.03 (0.36-2.91)	.96
Secondary education	40	0.90 (0.27-3.03)	.87
**Occupation status**			
Un-employed	98	1	
Business	66	0.58 (0.28-1.20)	.14
Employed	23	0.22 (0.06-0.74)	.01
**Marital status**			
Married/ cohabiting	109		
Single/ divorced	44		
Widow	34		
**Age at first intercourse**			
12-18	57	1	
19 or more	130	1.77 (0.83-3.74)	.140
**HIV**			
Negative	126	1	
Positive	61	0.66 (0.31-1.37)	.26
**Treatment given**			
Cryotherapy/thermal ablation	116		
LEEP	71	0.46 (0.21-0.99)	.04
**Days elapsed from screening to treatment**			
Single visit approach	82	1	
See and treat approach	105	0.83(0.37-1.85)	.65

Abbreviations: AOR, adjusted odd’s ratio;(where < 1 = less odds > 1 = more odds); HIV, human immune deficiency syndrome; LEEP, loop electrosurgical excision procedure; USD:United States Dollar.

### Barriers to adherence to follow-up from the health professionals’ perspective

During the IDIs, we identified various replies to the question: “What are barriers for women adhering to the follow-up recommendation after treatment for a precancerous lesion?” The responses were coded and subsequently grouped into themes and categories (Table 3).

**Table 3.** Themes and categories identified in the in-depth interviews.

Themes	Categories
**Low level of awareness**	Low level of understanding and lack of education
	Feeling healthy
	Thinking “Complete treatment”
	Forgetting
	Stigma related
**Barriers for reaching the clinic**	Long distance
	Transportation cost
	Travel and migration
	Late clinic arrival
**Personal barriers**	Local medicines
	Not liking the experience/not ready to expose private parts
	Male gender
	Have no time
**Barriers of service at the Health Facility**	Scarcity of health service providers
	Inadequate counselling by service providers
	Late reappointment due to pregnancy
	Shortage of equipment
	Lack of allowances
	Lack of a separate clinic
**Lack of reminders**	Unreachable phone numbers
	Lack of vouchers

#### Low level of awareness about the need for follow-up

Some of the healthcare providers mentioned a low level of understanding and a lack of education as barriers to adherence to follow-up. Often, this was linked to the absence of symptoms in the SCL. For instance, one healthcare provider stated: “*The community still lacks sufficient understanding because these lesions show no symptoms. […] If they were educated all the time, they would understand that they need to get screened even if they have no symptoms.”* Adding to that, another healthcare provider insisted: *“Education plays a significant role because people have different levels of understanding. You may tell them about the importance of follow-up in medical treatment, but they may not grasp it.”* Another mentioned the trouble of explaining why follow-up is needed: “*…They think what they have received is a complete treatment.”* Many suspected that low awareness increases the likelihood of forgetting about the follow-up: *“Sometimes, you call them, and they say they will come but then keep postponing. When you ask them later why they didn’t come, they might say they had to travel or simply forgot.”*

Some women had stigma relating to their HIV status, and this could affect their adherence, as well. One healthcare provider was quoted as describing difficulties while she tried to remind a client from an HIV care and treatment clinic: “*Those who trouble us are from HIV Care and Treatment Clinic (CTC), and when you call, they might say they don’t know you, and you have to inform them that you were given the number by someone else. Later on, they might say they didn’t want to talk or they were in a place where they couldn’t speak.”*

#### Barriers to reaching the clinic

Another frequently mentioned barrier to follow-up was the cost of transportation. One healthcare provider stated: *“The main contributing factor is the cost, money, to be precise. Many people can’t afford the cost of getting to the treatment centres from where they live.” Th*is is often related to the relatively long distance between home and health facility, as one health care provider claimed: “*The major challenge is that they lack transportation or say they are far away. You find that many come from distances of 5, 10, or even 20 kilometres.”* Another added: *“The main reason …, I think, is the distance. You can call someone, and they say they will come, but perhaps they don’t have the fare to get here.”* Additionally, some health care providers stated that patients had traveled or migrated after the initial screening and hence were unable to attend the follow-up screening: *“What I see [as] the biggest challenge is that customers sometimes move, they move from Boma, others come for business, so when they come across announcements there, they come; we test them, we find them, and we treat them. At the same time, she returns to her place of origin where she came from, so finding her again becomes difficult.”* Other healthcare providers described clients skipping or delaying follow-up visits because of job or business-related issues: “*They say that I don’t have time. I am at work.”*

#### Personal barriers

Another described barrier was the preference for traditional medicines. A health provider shared: *“Most women do not return, and [they] prefer going to traditional medicine providers after.”* One healthcare worker emphasized that some patients perceived themselves as healed after those visits: *“Others will say they have already been to a traditional healer and are using traditional remedies, so they are doing well.”*

#### Barriers at the health facility

Hindering adherence at the health facility was the scarcity of healthcare providers. One health professional explained: *“The staff who have received training are few, so when he is on vacation …, the service is unavailable. The staff who received training are few but still do other jobs, as well.”* Other health care providers stressed the need for training more staff to improve care: *“Another suggestion is to provide more training for other staff members so that those with skills become more numerous. Currently, we don’t have enough staff, which means that if we increase knowledge among more people, it will be easier to reach others.”*

Some healthcare providers recommended an allowance. One reported: *“If we health providers are at least well supported in terms of extra allowances, we would be able to trace these women. The allowance would be for tracing these women and encouraging them to return.”*

Another barrier to adherence was inadequate counseling. One health provider suggested that some healthcare providers do not counsel their clients properly, leading to client default at follow-up. “*Some are better at explaining to the client and emphasizing the importance of returning for check-ups, while others may not stress it enough. So, the quality of education provided by service providers is inconsistent.”*

#### Reminders

Some healthcare providers traced clients by calling them but frequently faced the issue that numbers were inactive, and hence those clients missed their follow-up visits. A health care provider said: “*Other challenges are they give you phone numbers, but later when they change them, you can’t find them again.”* Another barrier was a lack of credit or vouchers by the health care providers. One said: “*At the centre, I can say that sometimes I might not have sufficient airtime to call them, and we don’t get support from the centre to say that we will be given airtime vouchers.”*

### Factors associated with persistence or recurrent cervical lesions during follow-up screening

After adjusting for other factors, age was the only variable significantly associated with the persistence/recurrence of cervical lesions. Compared to young women (18-29 years), mature women aged 30-50 years had lower odds of persistence or recurrence of a cervical lesion (AOR = 0.21; 95% CI [0.05-0.91]), and post-menopausal women (>50 years) were less likely to have a persistent/recurrent cervical lesion (AOR = 0.14; 95% CI [0.02-0.85]) (Table 4).

**Table 4.** Factors associated with persistence/recurrent cervical lesion during follow-up screening (*n* = 130).

Variable	Total	Adjusted	
		AOR(95%CI) persistent/recurrent lesion	*P*-value
**Age**			
18-29	11	1	
30-50	90	0.16 (0.03-0.78)	.02
> 50	29		.16
**Marital status**			
Married/ cohabiting	81	1	
Single/ divorced	25	1.14 (0.32-4.05)	.84
Widow	24	0.57 (0.03-2.08)	.19
**Parity**			
0	5	1	
1-3	65	0.36 (0.06-2.02)	.24
4-5	47	0.85 (0.16-4.55)	.85
6 or more	13	0.29 (0.02-5.25)	.40
**Age at first sex**			
12-18	40	1	
19 or more	90	1.61(0.43-5.94)	.48
**HIV**			
Negative	85	1	
Positive	45	0.73(0.21-2.61)	.62
**Treatment type**			
Cryotherapy/thermo ablation	94	1	
LEEP	36	0.33(0.07-1.60)	.17

Abbreviations: AOR, adjusted odd’s ratio (where < 1 = less odds > 1 = more odds); HIV, human immune deficiency syndrome; LEEP, loop electrosurgical excision procedure.

## Discussion

This study assessed the adherence of women, treated for a SCL to the recommended follow-up screening after 1 year. An adherence rate of 65.3% was found, which is slightly higher in comparison to 2 studies done in Ethiopia, which reported adherence rates of 51% and 44.7%.^7,9^ Interestingly, most of the interviewed healthcare providers stated that the level of perception toward adherence after treatment was good. Other studies had shown the percentage of adherence to be between 38% and 75%, with those with higher rates employing more strategies (eg, reminder phone calls or text messages) to enhance adherence in the subsequent, planned follow-up.^10,11^ In this study, the high level of adherence generally could be attributed to the recruitment at a large tertiary teaching hospital. Possibly in these tertiary centres, patients are more likely to meet more doctors who could explain to them the fundamental reasons for why they should return.

### Barriers to follow-up examinations

Concerning the factors affecting adherence, reporting many individual barriers, being employed, and treatment by LEEP were factors associated with poor patient adherence. Our qualitative findings allowed deeper insights into the individual barriers, for example, the impact of long and costly journeys between home and health facility was stressed. Similarly, a cross-sectional study in Nigeria showed that those residing more than 10 km away from the facility were less likely to adhere to the recommended follow-up.^12^

Interestingly, we found that women who were employed were less likely to adhere to screening recommendations, similar to the findings of a study in Thailand.^13^ This may seem counter-intuitive as employed women might often have a higher education and more money available. However, health workers in the IDIs stressed that it is often difficult to take time off work to attend the screening.

Another individual barrier discussed in other qualitative research is fear.^8,14^ In our study, fear did not occur as a dominant topic in the IDIs. Still, it is one possible explanation for the finding that women treated with LEEP were less likely to adhere to follow-up compared to those treated with cryotherapy. Unlike cryotherapy, LEEP requires local anesthesia and a trained doctor. These are often unavailable and require a future visit, all can bring additional stress to the patient. We were unable to find a study that assessed follow-up adherence after LEEP compared to cryotherapy, but it might be a factor to bear in mind for future research.

Instead, healthcare workers expressed concerns that patients sought help from traditional healers and then perceived themselves as healed not seeing the need for further cervical cancer screenings.

It is known that patients tend to forget about follow-up appointments—this aspect was also stressed in the study in Ethiopia (Stroetmann et al, 2024). Therefore, it has been shown that reminders, for example via phone call can help to improve follow-up adherence.^8,11^ Until now, there is no standard reminder system for following up women with a SCL after treatment that established in Tanzania, but some healthcare workers explained that they tried already to call women in need of follow-up. They described that this is often difficult due to unreachable phone numbers. This observation is very much reflected in our own experience as only 54.8% of all women treated for SCL could be reached via phone call.

### Recurrence of cervical lesions

Of the participants who returned for follow-up screening, 17% were still positive for suspicious cervical lesions. This finding is similar to other studies that reported a 17.6% recurrence or persistence of an SCL following LEEP, and a 20% recurrence rate following ablative treatment (cryotherapy, laser ablation, and electrocauterization).^15,16^ The found recurrence or persistence rate stresses the importance of follow-up for those treated with precancerous lesions.

HIV status did not affect the recurrence or persistence of the lesion in this study. According to the WHO, sufficient data on the effect of HIV on the rate of SCL at rescreening is still lacking; some studies have reported higher rates in women living with HIV, while others have found no effect, in line with our study.^3,17^ Since most of our HIV patients were recruited at a tertiary hospital, we hope that a large majority were receiving sufficient treatment. However, we did not assess this factor (viral load).

Older women (ie, those greater or equal to 30 years old) had lower odds of persistence/recurrence of a cervical lesion compared to younger women. Similar findings were reported from studies in Nigeria and Taiwan.^18,19^

### Strengths and limitations

This is one of the first studies in East Africa to investigate adherence to follow-up after treatment for an SCL. We identified several factors limiting adherence through a combination of quantitative and qualitative methods and revealed high rates of recurrent or persistent cervical lesions, underscoring the need to address these barriers.

As we used secondary data from screening registry books, missing documentation and phone numbers and miss-entries were present. Also, some important predisposing factors for persistence or recurrence could not be assessed because of the lack of other important screening tests in health facilities, like HPV-DNA tests. This study researched the combined effects of the treatment options in the light of recurrence or persistence, unlike other studies where the outcomes of each treatment procedure were researched separately.

## Conclusion

One in 8 women who had a SCL during screening had a recurrent suspicious finding; this underscores the need to support the annual re-screening of these high-risk patients. Still, only 2 out of 3 women adhered to recommended follow-up. Individual factors such as “not knowing the need to return” and challenges of reaching the health facility were the most important drivers of non-adherence. Interventional studies to address these barriers perhaps including patient-centered counseling and addressing the socio-economic problems and use of reminder phone calls or text messages are needed to improve the situation in Tanzania. Given the huge, recent efforts and resources from governmental and non-governmental institutions leveraging primary screening across the continent, our results certainly emphasize a need for similar efforts to assure the re-screening of identified high-risk groups.

## Supplementary Material

oyaf111_suppl_Supplementary_Tables_S1

oyaf111_suppl_Supplementary_Tables_S2

## Data Availability

The data underlying this article cannot be shared publicly due to the privacy of individuals that participated in the study. The data shall be shared on a reasonable request to the corresponding author.
