# Supplementary material for: Barriers to adherence to cervical cancer screening care in Northern Tanzania
Source: Oncologist. 2025 May 16;30(5):oyaf111. doi: 10.1093/oncolo/oyaf111 (PMC12082816; doi:10.1093/oncolo/oyaf111)
Supplement: oyaf111_suppl_Supplementary_Tables_S1 [file oyaf111_suppl_supplementary_tables_s1.docx]

Supplemental Table S1.

| **INDIVIDUAL BARRIERS** | **SCORE** |
| --- | --- |
| Fearing an adverse outcome | +1 |
| Fearing the screening procedure | +1 |
| Not knowing the need for follow-up | +1 |
| Forgetting the follow-up appointment | +1 |
| Not reaching the health facility easily | +1 |
| Difficulty affording transportation costs | +1 |
| Not having time for attending follow-up | +1 |
| Male partner giving support | -0.5 |
| Receiving traditional treatment | +1 |
| Distance from home to the health facility > 5 km | +1 |
| Median score, individual barriers | 3.5 |
| **HEALTH FACILITY RELATED BARRIERS** | **SCORE** |
| Not receiving counselling | +1 |
| Not receiving counselling about the timing of follow-up | +1 |
| Having to wait a moderately long time | +0.5 |
| Having to wait a long time | +0.5 |
| Being unhappy with staff behaviour | +0.5 |
| Experiencing unavailability of the service | +1 |
| Challenges   - Shortage of heath commodities - Shortage of staff - Long wait time - Lack of privacy - Bad attitude of the staff - Other challenges | +1  +0.5  +05  +0.5  +1  +1 |
| Median score, health facility related barriers | 0.5 |
