# Supplementary material for: Barriers to adherence to cervical cancer screening care in Northern Tanzania
Source: Oncologist. 2025 May 16;30(5):oyaf111. doi: 10.1093/oncolo/oyaf111 (PMC12082816; doi:10.1093/oncolo/oyaf111)
Supplement: oyaf111_suppl_Supplementary_Tables_S2 [file oyaf111_suppl_supplementary_tables_s2.docx]

Supplemental Table S2.

**Barriers to follow-up adherence mentioned by patients in phone interviews**

| **Individual related barriers** | Frequency | Percent (%) |
| --- | --- | --- |
| **Fearing outcome** |  |  |
| Yes | 169 | 77.2 |
| No | 50 | 22.8 |
| **Fearing procedure** |  |  |
| No | 154 | 70.3 |
| Yes | 65 | 29.7 |
| **Not knowing the need to follow-up** |  |  |
| Yes | 194 | 90.2 |
| No | 21 | 9.8 |
| **Forgetting the follow-up** |  |  |
| Yes | 167 | 76.3 |
| No | 52 | 23.7 |
| **Not easy to reach the HF** |  |  |
| No | 180 | 82.2 |
| Yes | 39 | 17.8 |
| **Can afford the transport cost** |  |  |
| Yes | 182 | 83.1 |
| No | 37 | 16.9 |
| **Not having time** |  |  |
| No | 177 | 80.8 |
| Yes | 42 | 19.2 |
| **Partner support** |  |  |
| Yes | 214 | 97.7 |
| No | 5 | 2.3 |
| **Using traditional treatment** |  |  |
| No | 185 | 84.5 |
| Yes | 34 | 15.5 |
| **Distance to the HF** |  |  |
| <= 5 km | 53 | 25.9 |
| > 5 km | 151 | 74.0 |
| **Health facility related barriers** |  |  |
| Shortage of staff | 15 | 6.9 |
| Attitude of health care provider | 1 | 0.5 |
| Long waiting time | 12 | 5.5 |
| Lack of privacy | 1 | 0.5 |
| Limited commodities | 16 | 7.3 |
| Missed service at health facility | 16 | 7.31 |
| **Staff behaviour** |  |  |
| Satisfied | 172 | 78.5 |
| Not satisfied | 47 | 21.5 |
| **Waiting time** |  |  |
| Short time | 47 | 21.5 |
| Long time | 172 | 78.5 |
| **Told of the follow-up date** |  |  |
| Yes | 211 | 96.4 |
| No | 8 | 3.6 |
| **Counselled about the follow-up** |  |  |
| Yes | 214 | 97.7 |
| No | 5 | 2.3 |
